# Alcohol and other drug use by Australian workers: insights from a nationally representative cross-sectional survey

**DOI:** 10.1093/heapro/daaf048

**Published:** 2025-04-19

**Authors:** Gianluca Di Censo, Kirrilly Thompson, Jacqueline Bowden

**Affiliations:** National Centre for Education and Training on Addiction (NCETA), Flinders University, 4.10 Health Science Building, Bedford Park, South Australia, 5072, Australia; Flinders Health and Medical Research Institute, College of Medicine and Public Health, Flinders University, Bedford Park, South Australia, 5042, Australia; National Centre for Education and Training on Addiction (NCETA), Flinders University, 4.10 Health Science Building, Bedford Park, South Australia, 5072, Australia; Flinders Health and Medical Research Institute, College of Medicine and Public Health, Flinders University, Bedford Park, South Australia, 5042, Australia; National Centre for Education and Training on Addiction (NCETA), Flinders University, 4.10 Health Science Building, Bedford Park, South Australia, 5072, Australia; Flinders Health and Medical Research Institute, College of Medicine and Public Health, Flinders University, Bedford Park, South Australia, 5042, Australia

**Keywords:** workplace health, sector differences, occupation-related risk, Australian workforce, alcohol, illicit drug, tobacco

## Abstract

In the Australian population, a substantial number of people consume alcohol, tobacco, and other drugs (ATODs). As most people who use ATOD are employed, the workplace is an environment through which ATOD primary and secondary preventive health interventions can be implemented. However, resource allocation can be informed by research that determines priority industries and occupations, as indicated by the prevalence of ATOD use, numbers of users, and likelihood of use (i.e. adjusted odds ratios). A secondary analysis of the 2022–23 Australian National Drug Strategy Household Survey was conducted to assess risky alcohol consumption, current smoking, and illicit drug use (past 12 months) across different industries and occupations. There were 16,281 participants for industry analyses and 17,247 participants for occupation analyses. Survey-weighted estimations of the number and prevalence of individuals who consumed ATOD were performed, followed by a series of survey-weighted logistic regression analyses to identify industries and occupations associated with increased likelihood of ATOD use. Across all substances, the industries with the highest prevalence of ATOD use were mining, construction, and accommodation and food services, while occupations with the highest estimated prevalence were technicians and trades workers and labourers. Conversely, health care and social support, along with construction, exhibited the highest estimated number of ATOD users. The adjusted regression models revealed substantial variation in the industries and occupation types associated with heightened likelihood of ATOD use. This study provides important evidence for prioritizing industries and occupations most likely to benefit from ATOD interventions.

**Contribution to Health Promotion**

- Identifying workforce groups with the highest use of alcohol, tobacco, and other drugs (ATOD) is important to target workplace interventions and health promotion strategies.
- This study is the first to examine patterns of ATOD use across Australian industries and occupations by presenting estimates of prevalence and numbers of users, as well as adjusted regression models.
- Findings provide a comprehensive guide for prioritizing workplace interventions, providing essential information to aid decision-making among researchers, governments, policymakers, and health promotion practitioners in optimizing the allocation of limited public health resources and funding.

## INTRODUCTION

Over 14.5 million Australians, or 67.1% of the total population, were employed by the close of 2024 (Australian Bureau of Statistics 2024). As most Australians who use alcohol, tobacco, and other drugs (ATODs) are employed (McEntee et al. 2023), workplaces offer significant potential for the implementation of preventive policies and interventions aimed at reducing use and associated harms (Roman and Blum 2002, Ames and Bennett 2011, Morse et al. 2022). Common ATOD workplace interventions include policies, education and training, employee assistance programs, ATOD testing, health promotion, and screening and brief interventions (Duraisingam et al. 2022, Morse et al. 2022). Numerous studies indicate that workers in certain industries and occupations exhibit a greater likelihood of engaging in ATOD use (Berry et al. 2007, Marchand 2008, Roche et al. 2009, Pidd et al. 2011, Barnes and Brown 2012, Brown et al. 2015, Shockey and Esser 2020, McKetta et al. 2021, Pérez-Romero et al. 2023). This may result from numerous factors, such as the cultural norms of a workplace, an industry or occupation’s unique stressors, or the distinct demographics of people who are drawn to an industry or occupation type (Roche et al. 2015). The World Health Organization emphasize that workers’ health should be paramount to employers and recommends various strategies to support it, including health promotion initiatives (World Health Organization 2010). McEntee et al. (2023) assert that Australia has predominantly neglected the opportunity for workplaces to adopt cost-effective solutions to prevent and mitigate harmful ATOD use among the workforce. Furthermore, Australia’s Preventive Health Strategy highlights both the role that workplaces can play in facilitating or undermining good health and their potential as a settings-based approach for preventive health/health promotion interventions (Department of Health 2021). Often, such interventions require tailoring to specific sectors or workplaces to be effective and optimize return on investment (Duraisingam et al. 2022). Nonetheless, there could be more significant benefits from working with workforces that have larger numbers of ATOD users. A comprehensive examination of industries and occupations vulnerable to risky alcohol consumption, smoking, and illicit drug use, along with the total number of users, would inform resource allocation to maximize preventive health efforts and optimize population impact.

ATOD use within workplaces is complex. While certain industries and occupations may attract individuals who use different substances (Roche et al. 2015, Kaminski et al. 2019), work-related customs, practices, and conditions can also be associated with substance use, such as workplace subcultures, co-worker behavioural norms, dangerous work, and shift work (Pidd and Roche 2008). For example, a study by Roche et al. (2021) found that young people in construction demonstrated a heightened propensity for illicit drug use, which the authors attributed to their recent workforce integration and the resultant occupational pressures encountered early in their careers (i.e. stressful working conditions, harassment, demanding workloads, etc.). Some industries, such as mining and hospitality and food services, are characterized by excessive work hours (Heiler 2002, Murray‐Gibbons and Gibbons 2007, Kaminski et al. 2019) and shift arrangements that can lead to burnout and psychological and physical stress, which in turn can increase ATOD use (Pidd et al. 2014, Tynan et al. 2017, Rebar et al. 2018, Kaminski et al. 2019, Bufquin et al. 2021, James et al. 2021). Workplace stressors can lead to ATOD use either as a pre-emptive measure against anticipated stress (i.e. stress response dampening) or as a reaction to stressors encountered during work (i.e. stress-induced substance use) (Frone and Bamberger 2024). For instance, a worker in a physically demanding role might resort to non-prescribed opioids to manage the back pain caused by their job or may use them in preparation for an especially strenuous day at work.

Several studies have identified that workers in different industries and occupations exhibit a heightened propensity to engage in risky alcohol consumption. Two Australian studies conducted secondary analyses of the 2001 (Berry et al. 2007) and 2019 (McEntee et al. 2023) National Drug Household Surveys (NDSHS) and found that workers in the construction, agriculture, retail, manufacturing, and arts and recreation services industries had the highest prevalence of long-term risky alcohol consumption. Studies in the international literature typically yield similar findings, except for the observation that sectors, such as accommodation and food services (hospitality) and mining, exhibit a greater likelihood of engaging in risky alcohol consumption (Barnes and Brown 2012, Pulido et al. 2017, Ompad et al. 2019, Shockey and Esser 2020, Pérez-Romero et al. 2023). Regarding occupation, Berry et al. (2007) found that blue-collar workers had the highest prevalence of long-term risky alcohol consumption, while McEntee et al. (2023) identified managers and trades workers.

Several Australian studies have investigated the use of illicit drugs across various industries and occupational categories. Findings indicate that the construction, hospitality, and arts and recreational service sectors demonstrate the highest prevalence of use of any illicit drug (Bywood et al. 2006, Roche et al. 2009, 2022, McEntee et al. 2023). In addition, research indicates that blue-collar workers in trades or unskilled occupations demonstrate the highest prevalence of illicit substance use (Bywood et al. 2006, Roche et al. 2022, McEntee et al. 2023). In summary, the literature highlights several industries and occupations that are at risk of ATOD use. Although the Australian literature has not identified mining as an industry of particular concern, high rates of ATOD use in the mining industry have been identified in the international literature (e.g. Ompad et al. 2019, Shockey and Esser 2020, Pérez-Romero et al. 2023).

Identifying workforce groups most susceptible to ATOD use is crucial for targeting industries and occupations most likely to benefit from interventions (Morse et al. 2022). This information is significant for industry health and safety professionals, as well as public health researchers and policymakers, as it can inform specific ATOD workplace interventions and universal health promotion strategies aimed at reducing use and educating workers about harm reduction measures, thereby supporting the objectives of Australia’s National Drug (Department of Health 2017) and National Preventive Health strategies (Department of Health 2021). Most of the previous research on ATOD use by Australian workers has focused on the prevalence of use among industries and occupations (e.g. Bywood et al. 2006, Roche et al. 2009, 2022, McEntee et al. 2023). This gives a measure of the concentration of use, indicating the extent to which ATOD consumption is socially and culturally normalized in the workplace and among employees. The estimation of workforce population sizes is less common but is crucial for identifying the largest populations that would benefit from reduced substance use and could yield significant returns on investment from large-scale ATOD interventions. Finally, examining odds ratios is crucial for identifying industries and occupations that have workers with a heightened likelihood of ATOD use, especially when controlling for other associated variables. However, there has been no attempt to comprehensively discuss current patterns of ATOD use according to these multiple dimensions of prevalence, numbers, and odds ratios.

This study aims to analyse the most recent patterns in ATOD use in the Australian workforce, utilizing data from the 2022–23 NDSHS. The study provides estimates of the prevalence and numbers of ATOD users, along with odds ratios, to equip policymakers and health practitioners with evidence for the optimal allocation of resources aimed at reducing ATOD use and its associated harms. Therefore, the purpose of the current study was to explore patterns of risky alcohol consumption, tobacco use, and illicit drug use across Australian industries and occupations. The research questions guiding this study are: (1) Which industries and occupations have the highest prevalence of risky alcohol consumption, current tobacco use, and past-year illicit drug use? (2) Which industries and occupations account for the largest number of workers engaging in risky alcohol consumption, current tobacco use, and past-year illicit drug use? (3) Which industries and occupations exhibit the highest likelihood (odds ratios) of risky alcohol consumption, current tobacco use, and past-year illicit drug use?

## METHOD

### Participants

This study used data from the 2022–23 NDSHS survey, collected between 20 July 2022 and 31 May 2023. The NDSHS has been conducted biennially or triennially since 1985 and is the largest survey of a nationally representative sample of Australians aged 14 and older investigating alcohol and other drug use. The sampling method employed stratified, multistage random sampling. Data collection was completed using a multi-mode methodology (i.e. paper form, online form, or computer-assisted telephone interview). The final sample consisted of 21,663 participants. The application of person-level weights allowed for estimates to be obtained for the total Australian population aged 14 and older (*N* = 21,567,864). Weights were assigned to respondents in each stratum to account for selection probabilities, non-response, and discrepancies between the sample and the Australian population. Refer to the NDSHS Technical Report for information regarding the sampling and weighting procedure (Australian Institute of Health Welfare 2024).

The present analysis included only employed participants. Individuals who did not respond to the Australian and New Zealand Standard Industrial Classification (ANZSIC) question or whose industry was designated as ‘unclassifiable’ were omitted, resulting in a total of 16,281 participants for industry analyses. Individuals who did not respond to the Australian and New Zealand Standard Classification of Occupations (ANZSCO) question or whose occupation was categorized as ‘not in the workforce’ or ‘unclassifiable’ were omitted, resulting in a total of 17,247 participants for occupation analyses. Since the study is a secondary analysis of a publicly available dataset, ethics approval was not sought. However, the authors obtained permission from the custodians of the NDSHS data set to perform this analysis.

### Measures

The three dependent variables of interest in this study were alcohol consumption, current smoking status, and illicit drug use in the past 12 months. Risky alcohol consumption was measured in accordance with Australia’s Alcohol Guidelines set by the National Health and Medical Research Council (NHMRC), which recommends that adults consume no more than 10 standard alcohol beverages per week or no more than four on any one day (National Health and Medical Research Council 2020). Current smoking status was derived from a question asking if an individual currently, previously, or never smoked. This was recoded into a binary variable indicating current smoking status (no/yes). Past-year use of illicit drugs was a binary variable (no/yes) that indicated if a participant self-reported taking any illicit drug in the previous year. The two primary independent variables were industry and occupation type, following the NDSHS which uses the 19 ANZSIC industry categories (Australian Bureau of Statistics 2013) and 43 ANZSCO subgroups to classify occupation types (Australian Bureau of Statistics 2022). ANZSCO subgroups were recoded into the ANZSCO’s eight major occupation groups for analyses (see Table 1).

Regression models were adjusted to control for the variance of other variables. These variables were gender (male, female, or other), age, socioeconomic status (SEIFA, quantified in quintiles), educational attainment (year 11 or less [including certificate I or II], year 12, certificate II or IV, diploma, or bachelor’s degree or higher), marital status (never married, divorced/separated/widowed, married/de facto), whether they identified as Aboriginal and/or Torres Strait Islander (yes/no), birthplace in Australia (no/yes), rurality (major city, inner regional, or outer regional/remote), and weekly working hours. In addition, we adjusted for participants’ self-reported psychological distress scores in our models, which were measured using the Kessler 10 (K10) (Kessler et al. 2002).

### Statistical analysis

All statistical analyses were conducted using R (version 4.3.1) (R Core Team 2023) and the survey package (version 4.4.2) (Lumley 2024) to incorporate survey weights. The survey package was used to obtain estimates of the number and prevalence of individuals who engage in risky alcohol consumption, smoke tobacco, and use illicit drugs. We conducted regression models using svyglm with a quasibinomial family and a logit link function. The education and training industry category and the professional’s occupation category served as the reference groups for their respective models, as these categories had the lowest prevalence rates of ATOD use. We employed the Rao–Scott likelihood ratio test (Rao and Scott 1984) to assess if the adjusted models provided a better fit compared to the unadjusted models. We assessed the goodness-of-fit of the adjusted models using Tjur’s *R*^2^ (coefficient of discrimination), which is a measure of how well the predicted probabilities separate the outcome groups (Tjur 2009). We assessed how well the models discriminate between levels of the outcome variables using the area under the ROC curve (AUC). We fit unweighted models to screen for multicollinearity using the performance package (Lüdecke et al. 2021). No independent variables in the final models surpassed a variance inflation factor (VIF) of 10 (Midi et al. 2010). Moreover, we adjusted models for a range of covariates, including gender, age, socioeconomic status, educational attainment, marital status, indigeneity, birthplace in Australia, rurality, K10 score, and weekly working hours as these variables have been shown to be associated with use of ATOD (Wilsnack et al. 2002, Berry et al. 2007, Pidd et al. 2011, Inder et al. 2012, Tynan et al. 2017, Bufquin et al. 2021). We accepted a threshold for statistical significance of *P* < .05.

## RESULTS

### Industry prevalence and numbers of risky alcohol consumers, current smokers, and past-year illicit substance users

Seven different industry categories ranked amongst the top three for estimated prevalence (percentage) and total number of workers consuming alcohol at risky levels, currently smoking and using illicit substances in the previous year. As shown in Table 1, the highest prevalence of individuals consuming alcohol at risky levels was mining (53.1%), followed by construction (46.0%) and electricity, gas, water, and waste services (45.8%). However, the largest numbers of risky drinkers were in health care and social assistance (702,896), followed by construction (526,671) and education and training (505,125). The highest prevalence of current smoking was found in administrative and support services (31.7%), followed by mining (24.2%) and accommodation and food services (24.2%). However, the greatest number of current smokers was in health care and social support (235,184), followed by construction (153,194) and accommodation and food services (130,961). The highest prevalence of individuals using illicit drugs in the past year was accommodation and food services (27.6%), followed by construction (26.2%) and mining (24.3%). However, the largest numbers of users were in health care and social support (419,577), followed by accommodation and food services (320,540) and construction (280,712). Individuals employed in the education and training sector consistently ranked amongst the lowest two of all categories for prevalence of ATOD use, making them the reference group for subsequent regression models.

### Occupation prevalence and numbers of risky alcohol consumers, current smokers, and past-year illicit substance users

There was also notable heterogeneity amongst patterns of ATOD use across different occupation types (see Table 1). The highest prevalence of workers participating in risky alcohol consumption was technicians and trades workers (45.2%) and managers (40.7%), whereas professionals (1,317,909) and technicians and trades workers (919,824) had the largest numbers of risky drinkers. The highest prevalence of smoking tobacco was identified among labourers (33.0%) and machinery operators and drivers (28.3%), whereas the largest numbers of smokers were technicians and trades workers (262,937) and labourers (221,231). The highest prevalence of workers who used illicit drugs in the past year was among technicians and trades workers (24.7%) and labourers (21.7%), whereas the largest numbers of users were professionals (759,182) and technicians and trades workers (478,564). Since professionals consistently ranked among the lowest two occupations for the rates of ATOD use in terms of prevalence, they served as the reference group for subsequent regression models.

**Table 1.** Estimates of the total numbers and proportions of risky alcohol consumption, current smoking status, and past-year illicit drug use by industry and occupation type.

	Risky alcohol		Current smoking status		Past-year use of illicit drugs	
	*n* (95% CI)	% (95% CI)	*n* (95% CI)	% (95% CI)	*n* (95% CI)	% (95% CI)
**Industry**						
Agriculture, forestry and fishing	120,083 (89,851, 150,315)	40.6 (33.3, 48.3)	35,468 (21,438, 49,498)	22.9 (16.0, 31.0)	42,746 (24,341, 61,152)	15.1 (9.9, 21.4)
Mining	212,283 (170,582, 253,984)	53.1 (45.9, 60.3)	68,463 (46,882, 90,044)	24.2 (18.0, 31.3)	95,487 (70,571, 120,402)	24.3 (19.1, 30.1)
Manufacturing	315,175 (259,791, 370,559)	32.5 (27.7, 37.6)	108,030 (82,601, 133,459)	19.1 (15.5, 23.2)	152,536 (122,616, 182,456)	16.1 (13.3, 19.1)
Electricity, gas, water and waste services	88,601 (50,116, 127,087)	45.8 (33.2, 58.8)	19,977 (7,765, 32,189)	16.6 (9.0, 26.9)	36,472 (20,440, 52,504)	19.3 (12.6, 27.5)
Construction	526,671 (455,286, 598,056)	46.0 (40.9, 51.1)	153,194 (123,662, 182,726)	22.9 (19.3, 26.9)	280,712 (233,910, 327,515)	26.2 (22.6, 29.9)
Wholesale trade	93,972 (69,790, 118,154)	36.4 (28.7, 44.7)	41,126 (25,824, 56,428)	24.0 (17.2, 31.8)	59,234 (39,331, 79,137)	20.6 (15.0, 27.1)
Retail trade	410,091 (332,984, 487,199)	32.2 (27.5, 37.1)	130,369 (101,019, 159,719)	21.8 (18.0, 25.9)	253,417 (210,698, 296,136)	20.0 (17.1, 23.1)
Accommodation and food services	386,659 (312,154, 461,164)	31.9 (26.9, 37.3)	130,961 (100,196, 161,726)	24.2 (19.9, 28.9)	320,540 (268,386, 372,694)	27.6 (23.8, 31.6)
Transport, postal and warehousing	255,723 (204,642, 306,803)	32.1 (26.8, 37.7)	84,629 (64,699, 104,559)	19.5 (15.7, 23.8)	106,279 (80,824, 131,733)	15.0 (11.9, 18.5)
Information media and telecommunications	84,577 (46,138, 123,017)	39.8 (28.2, 52.3)	13,977 (6,277, 21,677)	10.2 (5.8, 16.2)	50,327 (33,891, 66,764)	20.7 (15.1, 27.1)
Financial and insurance services	205,834 (154,110, 257,557)	33.5 (26.9, 40.5)	38,718 (24,227, 53,208)	11.1 (7.7, 15.3)	121,068 (93,963, 148,174)	18.8 (15.2, 22.7)
Rental, hiring, and real estate services	66,343 (40,670, 92,016)	38.2 (26.4, 51.2)	11,651 (3,293, 20,009)	11.2 (5.0, 20.5)	27,973 (16,019, 39,927)	15.9 (10.2, 23.0)
Professional, scientific and technical services	385,674 (321,137, 450,210)	31.2 (26.8, 35.9)	67,359 (47,300, 87,417)	9.9 (7.5, 12.9)	241,139 (203,897, 278,381)	18.4 (15.9, 21.0)
Administrative and support services	194,309 (145,692, 242,926)	42.2 (35.0, 49.6)	86,269 (60,271, 112,266)	31.7 (24.8, 39.3)	98,040 (70,667, 125,414)	21.0 (16.2, 26.4)
Public administration and safety	308,353 (261,853, 354,853)	30.0 (25.8, 34.4)	74,367 (56,611, 92,122)	12.1 (9.6, 14.9)	141,598 (113,658, 169,538)	13.4 (11.1, 16.0)
Education and training	505,125 (423,578, 586,672)	27.4 (23.8, 31.3)	81,607 (59,882, 103,332)	9.1 (7.0, 11.5)	247,611 (209,875, 285,347)	13.8 (11.9, 15.8)
Health care and social assistance	702,896 (612,308, 793,485)	26.3 (23.4, 29.3)	235,184 (201,658, 268,711)	17.0 (14.9, 19.2)	419,577 (371,448, 467,707)	16.5 (14.8, 18.3)
Arts and recreation services	93,750 (52,036, 135,464)	33.7 (23.0, 45.7)	20,959 (11,952, 29,966)	14.5 (9.4, 20.8)	50,883 (34,498, 67,269)	19.0 (13.8, 25.1)
Other services	169,555 (131,077, 208,033)	31.6 (25.5, 38.2)	59,196 (38,757, 79,636)	19.1 (13.8, 25.3)	114,307 (85,860, 142,754)	23.2 (18.5, 28.3)
**Occupation**						
Managers	868,545 (770,056, 967,034)	40.7 (37.1, 44.3)	154,722 (128,317, 181,127)	11.6 (9.9, 13.6)	366,486 (322,067, 410,905)	17.1 (15.2, 19.0)
Professionals	1,317,909 (1,193,628, 1,442,190)	28.8 (26.5, 31.2)	205,159 (172,733, 237,585)	8.5 (7.3, 9.8)	759,182 (694,624, 823,740)	16.1 (14.9, 17.4)
Technicians and trades workers	919,824 (817,111, 1,022,537)	45.2 (41.5, 49.1)	262,937 (221,270, 304,603)	21.6 (18.7, 24.6)	478,564 (416,450, 540,677)	24.7 (22.1, 27.5)
Community and personal service workers	572,828 (491,407, 654,248)	30.0 (26.5, 33.7)	185,761 (155,414, 216,108)	19.3 (16.6, 22.1)	387,553 (334,045, 441,060)	20.6 (18.2, 23.2)
Clerical and administrative workers	628,607 (551,890, 705,323)	27.1 (24.2, 30.2)	203,516 (171,465, 235,568)	17.2 (14.9, 19.6)	340,683 (297,389, 383,977)	15.6 (13.8, 17.5)
Sales workers	333,740 (272,121, 395,359)	29.0 (24.6, 33.7)	120,425 (91,159, 149,690)	22.2 (18.1, 26.7)	254,695 (211,503, 297,886)	21.6 (18.4, 25.0)
Machinery operators and drivers	373,052 (307,938, 438,166)	36.2 (31.0, 41.6)	171,206 (137,527, 204,886)	28.3 (23.9, 33.0)	176,074 (140,313, 211,836)	19.9 (16.5, 23.6)
Labourers	408,903 (350,838, 466,967)	30.1 (26.3, 34.0)	221,231 (183,270, 259,192)	33.0 (28.9, 37.3)	279,035 (233,571, 324,499)	21.7 (18.7, 25.0)

### Models of ATOD use across industries

Binary logistic regression models were undertaken to examine the industries associated with increased odds of risky alcohol consumption, current smoking, and past-year illicit drug use, using education and training as the reference industry (see Table 2). In the unadjusted model, workers in seven industries were associated with increased odds of risky alcohol consumption. In adjusted models, only personnel in the administrative and support services (AOR 2.22, CI 1.28–3.85) and mining (AOR 2.15, CI 1.19–3.91) sectors were significantly more likely to partake in risky alcohol consumption. In the unadjusted model of current smoking, 11 different industries were associated with current smoking status. In the adjusted model, workers in administrative and support services (AOR 3.36, CI 1.67–6.78) and retail trade (AOR 1.72, CI 1.01–2.92) sectors were associated with increased odds of being a current smoker. In the unadjusted model of illicit drug use across industries, workers in 11 sectors exhibited increased odds of engaging in use. In the adjusted models, individuals in the electricity, gas, water, and waste services (AOR 2.18, CI 1.04–4.56), mining (AOR 2.17, CI 1.16–4.05), accommodation and food services (AOR 1.72, CI 1.19–2.49), and construction (AOR 1.63, CI 1.13–2.36) sectors exhibited statistically significant odds of partaking in illicit drug use.

**Table 2.** Unadjusted and adjusted binary logistic regression models of risky alcohol consumption, current smoking status, and past-year illicit drug use across industries.

	Alcohol		Tobacco		Illicit	
	OR (95% CI)	AOR (95% CI)	OR (95% CI)	AOR (95% CI)	OR (95% CI)	AOR (95% CI)
Industry (ref. education and training)	-	-	-	-	-	-
Agriculture, forestry and fishing	**1.81 (1.26, 2.61)** ^**^	1.01 (0.58, 1.75)	**2.98 (1.77, 5.03)** ^***^	1.21 (0.57, 2.59)	1.11 (0.68, 1.79)	1.08 (0.52, 2.27)
Mining	**3.00 (2.13, 4.24)** ^***^	**2.15 (1.19, 3.91)** ^*^	**3.20 (2.03, 5.04)** ^***^	1.61 (0.74, 3.51)	**2.00 (1.43, 2.81)** ^***^	**2.17 (1.16, 4.05)** ^*^
Manufacturing	1.27 (0.95, 1.71)	1.06 (0.69, 1.62)	**2.37 (1.62, 3.46)** ^***^	1.26 (0.72, 2.18)	1.20 (0.91, 1.57)	1.00 (0.68, 1.48)
Electricity, gas, water and waste services	**2.24 (1.29, 3.88)** ^**^	1.65 (0.78, 3.49)	2.00 (0.99, 4.06)	1.66 (0.54, 5.12)	1.49 (0.90, 2.47)	**2.18 (1.04, 4.56)** ^*^
Construction	**2.25 (1.71, 2.98)** ^***^	1.38 (0.90, 2.13)	**2.98 (2.09, 4.26)** ^***^	1.64 (0.96, 2.82)	**2.21 (1.72, 2.84)** ^***^	**1.63 (1.13, 2.36)** ^**^
Wholesale trade	**1.52 (1.02, 2.25)** ^*^	0.99 (0.55, 1.80)	**3.16 (1.91, 5.20)** ^***^	1.79 (0.81, 3.94)	**1.62 (1.08, 2.43)** ^*^	1.40 (0.74, 2.63)
Retail trade	1.26 (0.94, 1.68)	1.33 (0.84, 2.08)	**2.79 (1.92, 4.05)** ^***^	**1.72 (1.02, 2.92)** ^*^	**1.56 (1.22, 2.00)** ^***^	0.94 (0.65, 1.35)
Accommodation and food services	1.24 (0.92, 1.68)	1.27 (0.78, 2.07)	**3.19 (2.17, 4.70)** ^***^	1.62 (0.92, 2.84)	**2.38 (1.85, 3.07)** ^***^	**1.72 (1.19, 2.49)** ^**^
Transport, postal and warehousing	1.25 (0.91, 1.71)	0.85 (0.54, 1.34)	**2.43 (1.66, 3.57)** ^***^	1.41 (0.81, 2.46)	1.10 (0.81, 1.49)	0.96 (0.61, 1.49)
Information media and telecommunications	**1.75 (1.02, 3.01)** ^*^	1.62 (0.82, 3.23)	1.13 (0.59, 2.17)	1.01 (0.46, 2.22)	**1.63 (1.09, 2.43)** ^*^	1.62 (0.95, 2.75)
Financial and insurance services	1.33 (0.93, 1.91)	1.18 (0.70, 1.98)	1.25 (0.77, 2.03)	0.87 (0.47, 1.64)	**1.44 (1.07, 1.94)** ^*^	1.25 (0.84, 1.85)
Rental, hiring and real estate services	1.64 (0.94, 2.86)	0.90 (0.41, 1.97)	1.26 (0.56, 2.85)	1.03 (0.33, 3.28)	1.18 (0.71, 1.95)	1.28 (0.57, 2.87)
Professional, scientific and technical services	1.20 (0.90, 1.60)	0.96 (0.63, 1.46)	1.11 (0.73, 1.68)	1.22 (0.70, 2.12)	**1.40 (1.11, 1.78)** ^**^	1.26 (0.92, 1.74)
Administrative and support services	**1.93 (1.35, 2.76)** ^***^	**2.22 (1.28, 3.85)** ^**^	**4.65 (2.99, 7.25)** ^***^	**3.36 (1.67, 6.78)** ^**^	**1.66 (1.17, 2.35)** ^**^	1.60 (0.91, 2.81)
Public administration and safety	1.13 (0.86, 1.50)	0.86 (0.56, 1.30)	1.38 (0.95, 2.01)	0.89 (0.53, 1.49)	0.96 (0.74, 1.26)	0.86 (0.59, 1.26)
Health care and social assistance	0.94 (0.74, 1.20)	1.01 (0.69, 1.48)	**2.05 (1.49, 2.82)** ^***^	1.51 (0.97, 2.34)	**1.23 (1.00, 1.52)** ^*^	1.27 (0.95, 1.70)
Arts and recreation services	1.35 (0.78, 2.32)	1.08 (0.52, 2.25)	1.70 (0.97, 2.96)	0.66 (0.27, 1.59)	1.47 (0.98, 2.19)	0.77 (0.41, 1.43)
Other services	1.22 (0.87, 1.73)	0.74 (0.43, 1.27)	**2.37 (1.49, 3.78)** ^***^	0.78 (0.37, 1.65)	**1.88 (1.37, 2.59)** ^***^	1.44 (0.89, 2.34)
Rao–Scott LRT	2 × log LR = 288.51, *p* < 0.001		2 × log LR = 277.23, *p* < 0.001		2 × log LR = 657.49, *p* < 0.001	
AUC	0.67		0.72		0.75	
Tjur’s *R*²	0.09		0.10		0.13	

Notes:

1. OR = Odds ratio, AOR = Adjusted odds ratio.

2. ^*^P < .05, ^**^P < .01, ^***^P < .001.

3. Adjusted for gender, age, socioeconomic status, educational attainment, marital status, indigeneity, born in Australia, rurality, K10 score, and hours worked per week.

### Models of ATOD use across occupations

Binary logistic regression models were undertaken to examine the occupations associated with increased odds of risky alcohol consumption, current smoking, and past-year illicit drug use, using professionals as the reference group (see Table 3). In the unadjusted model of risky alcohol consumption, technicians and trades workers (OR 2.04, CI 1.69–2.47), managers (OR 1.70, CI 1.41–2.05), and machinery operators and drivers (OR 1.40, CI 1.08–1.81) exhibited elevated odds of risky alcohol consumption. In the adjusted model, only personnel in management positions (AOR 1.50, CI 1.13–1.99) were associated with increased odds of risky alcohol consumption. In the unadjusted model of current smoking, all eight occupational categories were significantly associated with current smoking status. In the adjusted model, only labourers (AOR 2.20, CI 1.46–3.30) and machinery operators and drivers (AOR 1.67, CI 1.04–2.68) were significantly associated with increased odds of being a current smoker. In the unadjusted model of illicit drug use, five occupational types exhibited significantly elevated odds ratios. In the adjusted models, only technicians and trades workers (AOR 1.40, CI 1.04–1.89) were associated with increased odds of illicit substance use.

**Table 3.** Unadjusted and adjusted binary logistic regression models of risky alcohol consumption, current smoking status, and past-year illicit drug use across occupations.

	Alcohol		Tobacco		Illicit	
	OR (95% CI)	AOR (95% CI)	OR (95% CI)	AOR (95% CI)	OR (95% CI)	AOR (95% CI)
Occupation (ref. professionals)	-	-	-	-	-	-
Managers	**1.70 (1.41, 2.05)** ^***^	**1.50 (1.13, 1.99)** ^**^	**1.42 (1.11, 1.82)** ^**^	1.15 (0.83, 1.61)	1.07 (0.91, 1.26)	1.08 (0.86, 1.35)
Technicians and trades workers	**2.04 (1.69, 2.47)** ^***^	1.34 (0.96, 1.87)	**2.98 (2.33, 3.80)** ^***^	1.38 (0.91, 2.09)	**1.71 (1.44, 2.04)** ^***^	**1.40 (1.04, 1.89)** ^*^
Community and personal service workers	1.06 (0.86, 1.30)	1.15 (0.83, 1.59)	**2.58 (2.02, 3.31)** ^***^	1.20 (0.83, 1.73)	**1.35 (1.13, 1.62)** ^***^	1.02 (0.76, 1.37)
Clerical and administrative workers	0.92 (0.76, 1.11)	0.94 (0.68, 1.29)	**2.24 (1.77, 2.85)** ^***^	1.02 (0.72, 1.45)	0.96 (0.81, 1.14)	1.02 (0.79, 1.31)
Sales workers	1.01 (0.79, 1.29)	0.90 (0.60, 1.36)	**3.09 (2.25, 4.23)** ^***^	1.47 (0.93, 2.32)	**1.43 (1.16, 1.78)** ^***^	0.92 (0.66, 1.29)
Machinery operators and drivers	**1.40 (1.08, 1.81)** ^*^	0.89 (0.56, 1.40)	**4.27 (3.22, 5.66)** ^***^	**1.67 (1.04, 2.68)** ^*^	**1.29 (1.02, 1.65)** ^*^	1.26 (0.84, 1.89)
Labourers	1.06 (0.86, 1.32)	0.92 (0.64, 1.33)	**5.33 (4.09, 6.95)** ^***^	**2.20 (1.46, 3.30)** ^***^	**1.45 (1.17, 1.78)** ^***^	1.04 (0.71, 1.50)
Rao–Scott LRT	2 × log LR = 278.94, p < 0.001		2 × log LR = 266.43, p < 0.001		2 × log LR = 670.63, p < 0.001	
AUC	0.67		0.72		0.75	
Tjur’s *R*²	0.08		0.10		0.12	

Notes:

1. OR = Odds ratio, AOR = Adjusted odds ratio.

2. ^*^P < .05, ^**^P < .01, ^***^P < .001.

3. Adjusted for gender, age, socioeconomic status, educational attainment, marital status, indigeneity, born in Australia, rurality, K10 score, and hours worked per week.

## DISCUSSION

The purpose of the present study was to identify industries and occupations where workers exhibit elevated rates of ATOD use according to estimated prevalence and number of users, as well as odds ratios. Priority industries differ according to the perspective used to understand patterns of use. For risky alcohol consumption, the industries with the highest prevalence were mining and construction, while the largest numbers of workers were found in health care and social assistance and construction. For current smoking status, administrative and support services, along with mining, showed the highest prevalence, whereas healthcare and social support, as well as construction, had the largest estimated numbers of current smokers. The highest rates of past-year illicit drug use were found in accommodation and food services, as well as construction, while health care and social support, along with accommodation and food services, had the largest estimated numbers of users affected. From a prevalence standpoint, the mining and accommodation and food services sectors were identified as industries where ATOD workplace interventions may effectively target the most concentrated populations of ATOD users. On the other hand, when looking from a population perspective, health care and social support, along with construction, were identified as industries where workplace intervention could reach the largest number of ATOD users.

Similar distinctions were observed when comparing the estimated prevalence of ATOD use and estimated numbers of ATOD users across occupation types. For risky drinking and illicit drug use, technicians and trades workers demonstrated the highest prevalence, whereas professionals had the largest estimated numbers of risky drinkers and illicit drug users. For current smoking status, labourers exhibited the highest prevalence, whereas technicians and trades workers were identified as the occupation type with the largest numbers of current smokers. Consequently, when designing interventions for specific occupational groups, professionals and technicians, as well as trade workers, are identified as the most appropriate targets for reducing ATOD use. These findings about ATOD patterns by occupation highlight the need for ATOD interventions targeting multiple substances to be relevant to all levels of workers in organizations, including those in both ‘white collar’ and ‘blue collar’ occupational positions.

Adjusting for various other factors that contribute to ATOD provides insights into specific industries or occupation types with unique characteristics that contribute to ATOD use. According to adjusted analyses, employees in the mining and administrative and support services sectors, as well as those in management occupations, are more likely to exceed Australia’s Alcohol Guidelines (National Health and Medical Research Council 2020), putting themselves at increased short- and long-term harms. Additionally, individuals working in retail and administrative and support services, as well as machinery operators, exhibit the greatest likelihood of being current smokers. Individuals in the mining, electricity, gas, water and waste services, and accommodation and food services sectors, as well as technicians and trades workers, exhibited the highest odds of engaging in illicit drug use. These findings indicate that specific industries, including mining and accommodation and food services, may possess characteristics that attract individuals who use ATOD, may have environments that encourage use (e.g. availability, culture, etc.), or they may impose particular demands on their workforce that promote such use (e.g. shift work, occupational stress, availability of substances, etc.) (Pidd et al. 2014, Roche et al. 2015, Tynan et al. 2017, James et al. 2021, Frone and Bamberger 2024).

Our findings are consistent with prior research from both Australia (Roche et al. 2009, Pidd et al. 2011, 2014, Gates et al. 2014) and overseas (Pulido et al. 2017, Shockey and Esser 2020, Pérez-Romero et al. 2023) demonstrating elevated ATOD use in the construction and accommodation and food services sectors. However, our analysis identified the mining industry as particularly vulnerable to risky alcohol and illicit drug consumption. While this finding aligns with the international literature (Barnes and Brown 2012, Pulido et al. 2017, Ompad et al. 2019, Shockey and Esser 2020), it is not supported by prior Australian research (Bywood et al. 2006, Roche et al. 2009, Brown et al. 2015), suggesting a potential emerging trend. Nevertheless, it likely reflects the physically demanding nature of the mining sector, shift work, fly-in-fly-out rostering, the necessity for workers to be away from home (or, conversely, to take full advantage of time away from worksites where ATOD screening occurs), and cultures of risky drinking (Heiler 2002, Tynan et al. 2017, Rebar et al. 2018, James et al. 2021). Another novel finding of the present study was that individuals in the administrative and support services sector were at a particularly high risk of consuming alcohol in excess of the NHMRC guidelines and being current smokers. Our novel results could be attributed to our examination of use patterns following the COVID-19 pandemic, in contrast to studies that investigated use patterns prior to the pandemic. However, further research using longitudinal data is necessary to determine whether the COVID-19 pandemic significantly influenced ATOD use. Furthermore, our results align with literature that shows individuals in blue-collar and lower-skill occupations exhibit a greater likelihood to use illicit drugs (Bywood et al. 2006, Roche et al. 2009, Brown et al. 2015), while those in management and blue-collar positions are more likely to engage in risky alcohol consumption (Berry et al. 2007, Marchand 2008, Pidd et al. 2011, Barnes and Brown 2012, Shockey and Esser 2020).

This study provides decision-makers with insights on allocating the limited funds available for ATOD-related interventions, while other research has highlighted the most effective strategies. A systematic review of workplace-based interventions by Morse et al. (2022) demonstrated that general health promotion and screening and brief interventions were associated with a reduction in ATOD use. Additionally, the authors mention that screening (i.e. identifying workers who have substance use problems or are at risk of developing them), as an intervention in itself, was associated with reductions in ATOD use. Lee et al. (2014) argue that ATOD screening alone is insufficient in industries predominantly occupied by males, such as mining. Therefore, this indicates the necessity for multi-faceted interventions encompassing policy, education, brief interventions, and access to counselling, which should be incorporated into a comprehensive health promotion strategy centred on health and wellbeing, as suggested by Duraisingam et al. (2022). This highlights the necessity of collaborating with the industries and occupation types identified in this study to co-design interventions that are tailored to specific workplaces, as workplaces have unique characteristics that must be taken into account when developing such interventions (e.g. workplace demands and stressors).

### Limitations

The present study has several limitations. As noted earlier, different industries and occupations present different workplace demands and stressors, which could potentially promote the use of ATOD (Murray‐Gibbons and Gibbons 2007, Pidd et al. 2014, Frone 2015, Roche et al. 2015, Morse et al. 2022, Frone and Bamberger 2024). For instance, individuals in certain sectors may experience elevated levels of burnout and a higher frequency of workplace harassment, in addition to being physically or mentally taxed. However, these variables were not included in our analyses because they fall outside the scope of the NDSHS. Nevertheless, the methodology used in the present study was able to identify industries and occupations with an elevated likelihood of ATOD use.

Additional regression analyses indicated that individuals with missing industry data (*n* = 172) were more likely to consume alcohol at risky levels and use illicit drugs, though no significant difference was observed for smoking status. This suggests that some degree of selection bias may be present. The goodness-of-fit statistics indicated that our models exhibited only a moderate capacity to predict ATOD use. This could be attributed to our assessment of broad use categories (e.g. high-risk alcohol consumption and illicit drug use over the past year). It is possible that a focus on acute outcome variables, such as weekly use of illicit drugs, would have resulted in a better fit of our models with the data. Future research is necessary to determine these more immediate and concerning indicators of elevated harm. This would introduce an additional dimension for prioritizing workplace ATOD interventions that takes into account not just health-related consequences of use, such as disease and illness, but also occupational health and safety-related effects, such as intoxication during work hours. In addition, future research could explore the impact of socio-demographic, health, and behavioural characteristics associated with ATOD consumption among the workforce overall, as well as within specific industries and occupations identified in this study as having an increased risk of ATOD use.

### Conclusion

This study is the first to examine patterns of ATOD use across Australian industries and occupations by presenting estimates of prevalence and numbers of users, as well as adjusted regression models. Thus, it offers the most current, comprehensive, and nuanced prioritization tool for ATOD interventions in the workplace. This is important information for supporting the decision-making by researchers, governments, policymakers, and health promotion practitioners to maximize the use of limited public health resources and funding.

## Data Availability

The data underlying this article were provided by the Australian Institute of Health and Welfare. Data can be accessed on request at: https://doi.org/10.26193/U6LY7H.
